# Case Report: Hybrid approach as a Rescue Treatment in a patient with vascular Ehlers–Danlos Syndrome

**DOI:** 10.3389/fsurg.2023.1268671

**Published:** 2023-10-23

**Authors:** Suehyun Park, Deokbi Hwang, Woo-Sung Yun, Hyung-Kee Kim, Seung Huh

**Affiliations:** ^1^ Division of Vascular and Endovascular Surgery, Department of Surgery, Kyungpook National University Hospital, Kyungpook National University School of Medicine, Daegu, Republic of Korea; ^2^ Division of Vascular and Endovascular Surgery, Department of Surgery, Kyungpook National University Chilgok Hospital, School of Medicine, Kyungpook National University, Daegu, Republic of Korea

**Keywords:** iliac aneurysm, arteriovenous fistula, endovascular procedure complication, vascular closure devices, vascular Ehlers-Danlos syndrome (vEDS), vascular Ehlers-Danlos syndrome [type IV]

## Abstract

Vascular Ehlers-Danlos Syndrome (vEDS) is a rare connective tissue disorder associated with *COL3A1* gene mutation encoding type III collagen. Given the possible fatal prognosis if not treated timely, it is important to suspect and diagnose as soon as possible. Despite advances in endovascular technique, access point complications remain a serious challenge in patients with vEDS. Here, we describe a 30-year-old male patient who was diagnosed with vEDS after consecutive events of bilateral iliac vessels at an interval of 3 months: (1) spontaneous dissecting aneurysm of right iliac artery and (2) arteriovenous fistula between left internal iliac artery (IIA) and left common iliac vein. This patient was treated with iliac stent-grafts and overlapping femoral interposition graft (Dacron) in the 1st operation and access artery repair with surgical dissection after coil embolization of IIA and stent-graft insertion into left common to external iliac arteries in the 2nd operation. The patient has been treated with beta-blockers and anticoagulants for the management of vEDS and postoperative deep vein thrombosis, respectively. The stent-grafts in both iliac arteries and the access sites have been well-tolerated without any adverse effects for 14 months following the 2nd operation. In conclusion, given the vascular fragility and the potential for future events, additional vascular manipulation should be avoided unless it is in a life-threatening condition. In particular, meticulous hybrid interventions can be effective treatments.

## Introduction

Vascular Ehlers-Danlos Syndrome (vEDS) is usually inherited in an autosomal dominant manner. It is caused by mutations in the *COL3A1* gene encoding the pro-alpha-1 chains of type III collagen, which is a major protein of blood vessel walls and hollow organs (1, 2). Therefore, among the 6 major subtypes of EDS, vEDS is the most severe and catastrophic structurally, leading to unexpected arterial aneurysms or rupture, bowel perforation, uterine rupture at the time of delivery or sudden death at a young age and a life expectancy of less than 50 years on average (3). Thus, it is important to be aware of the characteristics of vEDS and suspect its occurrence as soon as possible because timely treatment can significantly influence the prognosis. Since the first report of successful stent-graft insertion in an iliac artery aneurysm in a patient with vEDS in 2006, the risk of access site complications as well as the feasibility of endovascular treatment in patients with vEDS have been reported steadily (4–6). Here, we describe a young male patient who was diagnosed with vEDS after metachronous lesions of bilateral iliac vessels and who was treated differently for the bilateral access sites following stent-graft placement. Patient's informed consent and approval of our Institutional Review Board (IRB # 2023-02-001-001) were obtained.

## Case presentation

**Figure 1:**
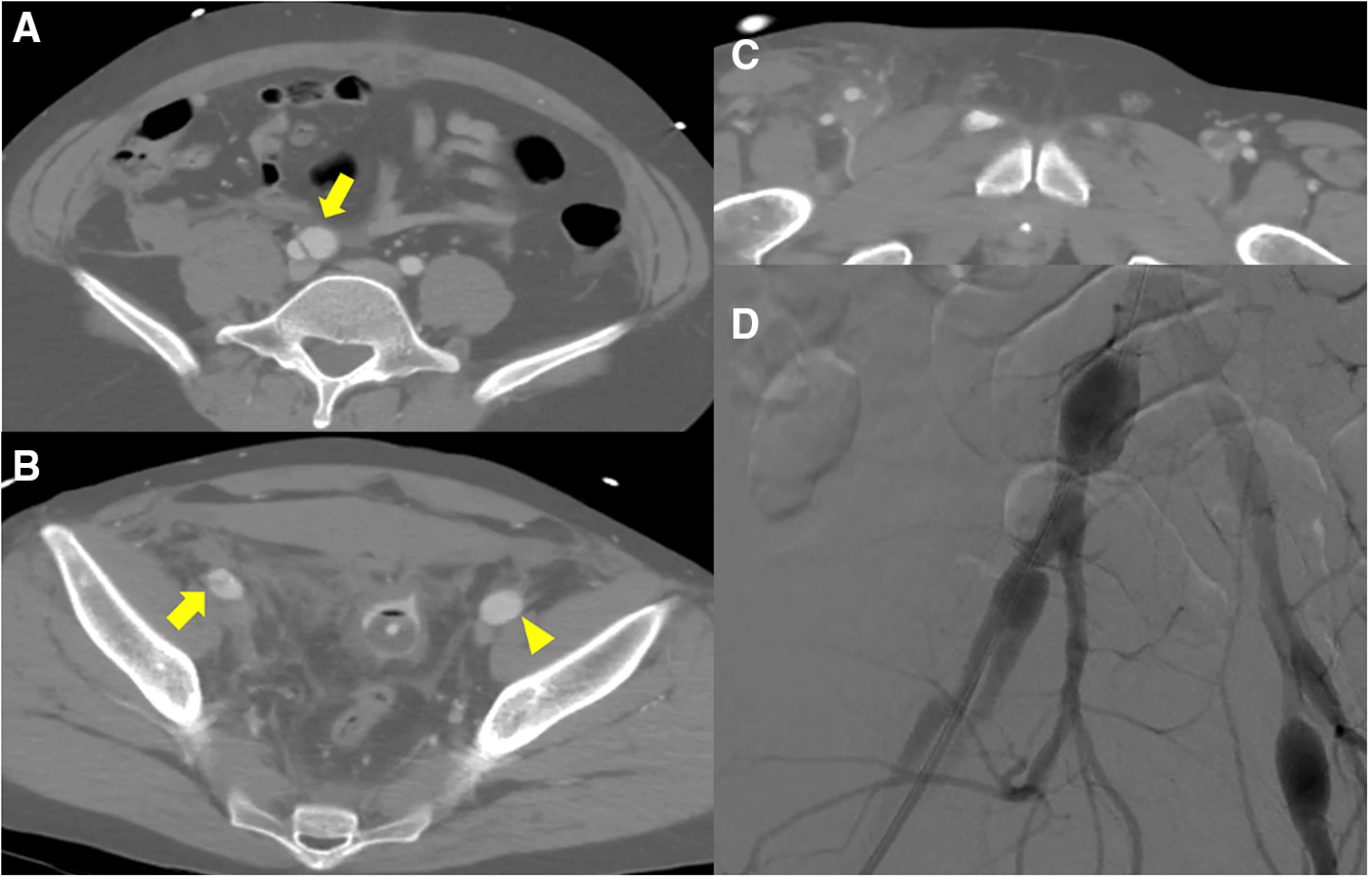
Initial computed tomography scan shows (**A**) dissecting aneurysms (*yellow arrow*) from right common iliac artery to (**B**) external iliac artery and aneurysmal dilatation of left external iliac artery (*yellow arrowhead*). (**B**, **C**) Hemoperitoneum was detected in the pelvic cavity and the area surrounding right superficial femoral artery; however, no active bleeding was detected. (**D**) Intraoperative fluoroscopic angiography shows pathological aneurysms on both sides.

**Figure 2:**
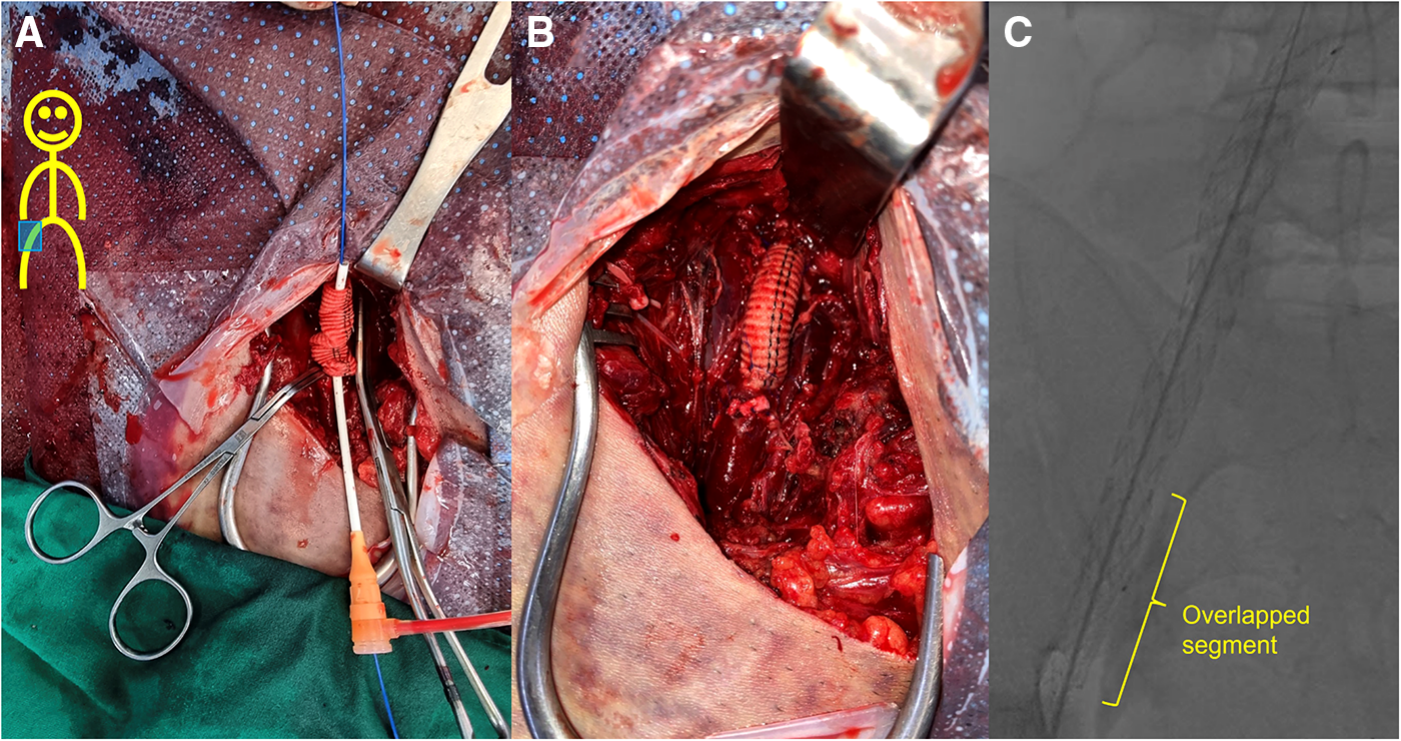
Field of the 1st operation. (**A**) After the 8-mm Dacron graft and the right common femoral artery were anastomosed using felt pledgets, the Dacron interposition graft was punctured near the distal anastomosis and a 7 Fr sheath and 0.035-inch guidewire were inserted. The Dacron graft was inserted to overlap the iliac stent-graft by approximately 5 cm in length while controlling inflow with an inflated balloon catheter along the guidewire that entered the iliac stent-graft. A 10 mm × 58 mm LifeStream™ balloon-expandable covered stent (Bard Peripheral Vascular, Tempe, AZ, USA) was then placed in the nested area for fixation. (**B**) After removal of sheath and before wound closure, reinforcement with 6-0 Prolene sutures was performed sparsely between the stent-graft of external iliac artery (EIA) and Dacron graft, which was inserted within the EIA stent-graft. (**C**) The final angiogram shows successful placement of the overlapping stent-grafts with no folds.

**Figure 3:**
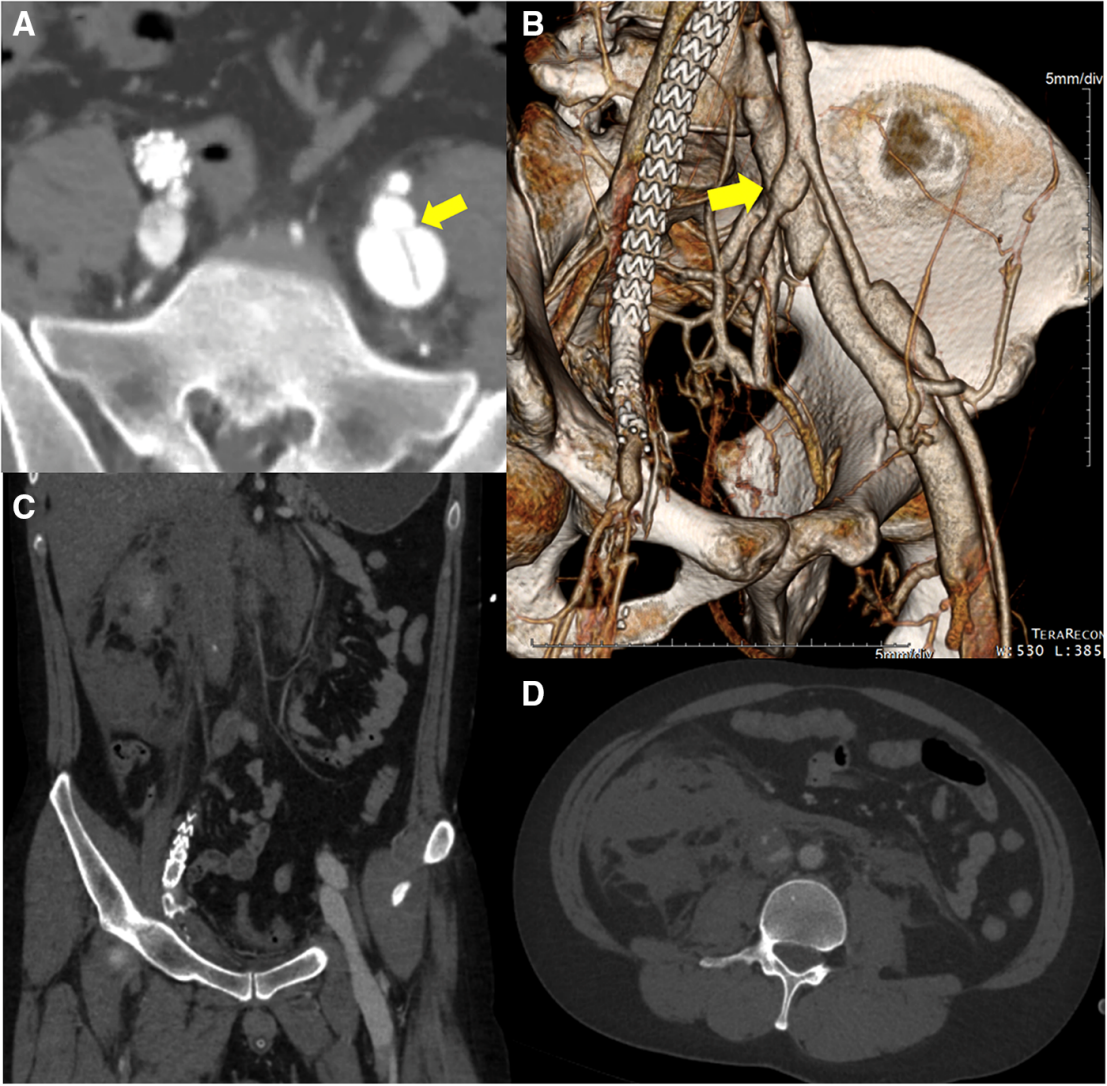
The initial computed tomography angiography before the 2nd operation reveals (**A**, **B**) an arteriovenous fistula (*yellow arrow*) between left internal iliac artery and left common iliac vein. Simultaneous contrast enhancement of arteries and veins is confirmed. (**C**, **D**) Large amount of hematoma near infrarenal inferior vena cava probably due to the intravenous high pressure generated by the iliac arteriovenous fistula and disease-specific vascular fragility.

**Figure 4:**
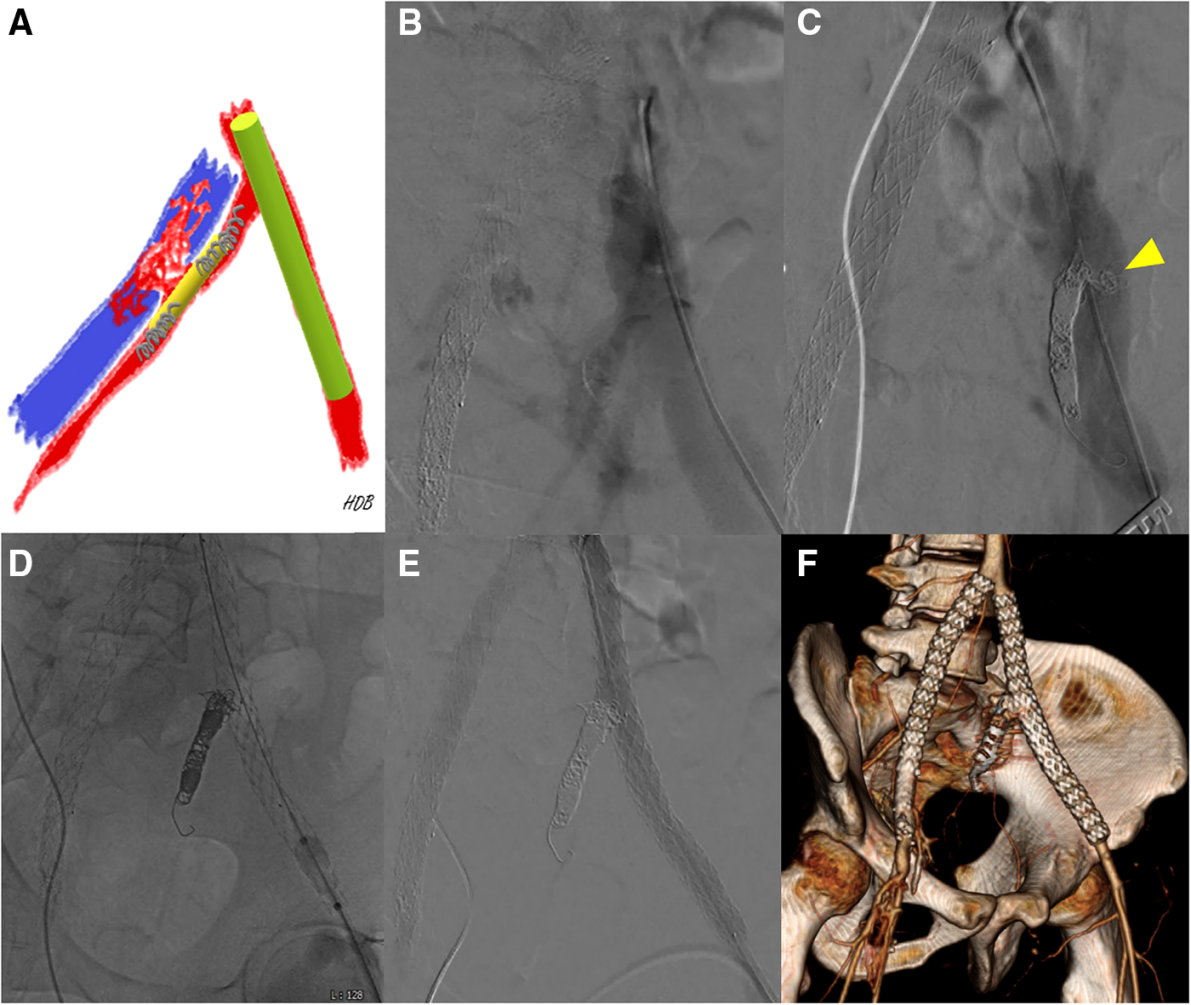
(**A**) A schematic diagram showing preoperative planning [red, left iliac artery; blue, left common iliac vein (CIV); yellow, self-expanding stent; light green, stent-graft; gray, microcoils]. (**B**) Initial intraoperative angiography shows simultaneous contrast filling in the left iliac artery and left CIV. (**C**) Even after coil embolization on both ends of the self-expanding stent, angiography shows contrast-filled left CIV. Proximal internal iliac artery before fistula was too short and the fistula was larger than expected. The yellow arrowhead indicates fluttering coils into the iliac vein through the fistula. (**D**) The 13 mm stent-grafts were placed across the left iliac arteries to block the flow through the fistula. (**E**) A final angiogram shows that patent stent-grafts in the iliac arteries without any flow through the fistula into the iliac veins. (**F**) On postoperative day 10, a CT scan revealed a decreased extent of intraabdominal hematoma with no signs of bleeding. Additionally, the scan showed calcinosis surrounding the right common femoral artery and external iliac artery, as well as well-patent iliac stent-grafts and successful embolization of the left internal iliac artery.

A 30-year-old male patient with no medical, familial or traumatic history was admitted to our emergency department with acute onset abdominal pain. Initial vital signs were normotensive (115/66 mmHg) but tachycardiac (115/min). Physical examination revealed abdominal tenderness in the right lower quadrant. The patient had a history of bruising easily since childhood and his skin was so lucent that the superficial vein was markedly visible (Supplementary Figure S1). An abdominal computed tomography (CT) scan revealed two dissecting aneurysms of right iliac arteries, along with an aneurysmal dilatation of the left external iliac artery (EIA). Hemoperitoneum was observed in the right groin and pelvic cavity, but there was no evidence of active bleeding (Figures 1A–C). Compared with initial blood tests, which were within normal range, follow-up tests showed a decrease in hemoglobin (13.4→10.7 g/dl). Due to concerns associated with ruptured iliac artery aneurysm, urgent operation was performed and intraoperative fluoroscopic angiography confirmed the CT findings (Figure 1D). Two pieces of stent-grafts (INCRAFT® 13 mm × 10 mm × 140 mm, Cordis Corp., Milpitas, CA, USA; COVERA™ Plus 10 mm × 60 mm, Bard, Tempe, USA) were deployed along the right iliac arteries, together with a 10 mm × 40 mm self-expanding stent (Absolute Pro®, Abbott Vascular, Santa Clara, CA, USA) up to the right EIA completely covering the aneurysm segment. When we advanced the initially preloaded knot of Perclose Proglide™ devices (Abbott Vascular, Redwood City, CA, USA) to complete the procedure, the blood vessel was injured, which required immediately incision in the right groin for surgical repair. The common femoral artery (CFA) was already torn and too friable to be repaired primarily. We decided to perform graft interposition across the torn CFA segment. First, a distal anastomosis was conducted between 8-mm Dacron graft and CFA using felt pledgets. However, proximal anastomosis was impossible due to insufficient clamping length and blood-stained tissues. The Dacron interposition graft was punctured near the distal anastomosis and a 7 Fr sheath and 0.035-inch guidewire were inserted. While controlling inflow with an inflated balloon catheter along the guidewire that entered the iliac stent-graft, we inserted the distally anastomosed interposition graft into the iliac stent-graft over the balloon catheter and deployed an additional balloon-expandable covered stent (LifeStream™; Bard Peripheral Vascular, Tempe, AZ, USA) inside the overlapped segment for fixation. After removal of sheath and before wound closure, reinforcement with 6-0 Prolene sutures was performed sparsely between the EIA stent-graft and Dacron graft, which was inserted within the EIA stent-graft. The final angiogram revealed no extravasation or stenotic occlusions (Figure 2). The patient was discharged on postoperative day 20. One month later, deep vein thrombosis (DVT) developed below the right common femoral vein. Anticoagulant was prescribed, which was switched from Aspirin to Xarelto. The patient was transferred to our hospital due to presyncope 3 months after the initial event. Initially, hypotension (95/64 mmHg) and tachycardia (131/min) were detected. A CT scan showed an arteriovenous fistula (AVF) between left internal iliac artery (IIA) and left common iliac vein (CIV), and a large amount of hematoma near infrarenal inferior vena cava (Figure 3). After admission, tachycardia continued and the left leg was swollen, bluish and painful in a single day. A duplex scan performed at the bedside suggested venous hypertension, but no DVT on the left leg. The follow-up CT showed no increase of hematoma. However, due to concerns of imminent heart failure and Phlegmasia cerulea dolens (PCD) by uncorrected AVF, we decided to perform an urgent operation via an endovascular approach. First, we deployed an 8 mm × 40 mm Everflex (Medtronic, Minneapolis, MN, USA) self-expanding stent within IIA to cover the opening of fistula and embolized distal IIA and proximal end of stent with microcoils to seal the AVF orifice. However, the opening of fistula was substantially larger and closer to iliac artery bifurcation than expected and the arterial flow was not completely sealed with coils, especially at the proximal end of the stent. To completely exclude AVF, two pieces of INCRAFT stent-grafts (13 mm × 13 mm × 120 mm, 13 mm × 13 mm × 100 mm) were deployed from left common iliac artery to EIA. The final angiogram revealed no leakage (Figures 4A–E). After completing the endovascular procedure, we incised the groin and surgically dissected the left CFA while keeping the sheath in place. After removing the sheath, the access artery was carefully repaired using Prolene 6-0 in a usual interrupted suture technique. Due to the vessel fragility observed in the previous operation, and especially the concerns regarding demanding bleeding control due to venous congestion caused by AVF, surgical repair of the CFA puncture site was pre-planned to be performed after the completion of the endovascular procedure. On postoperative day 10, DVT was newly diagnosed below the left mid CIV. On postoperative day 18, the patient was discharged with an anticoagulant prescription after confirmation of no further bleeding (Figure 4F). Due to repeated vascular events without identifiable cause, vascular fragility and history of easy bruising, we performed a genetic evaluation under the suspicion of connective tissue disease. Genetic assessment confirmed vEDS based on the detection of a pathogenic variant in the *COL3A1* gene: c.674G>A, heterozygous, p.(Gly225Asp). At four months after the second operation, the patient visited ER with right lower quadrant pain in the abdomen. Based on a diagnosis of acute appendicitis without perforation, laparoscopic appendectomy was performed without any complications. Fourteen months after the second operation, the patient is currently on a beta-blocker to control blood pressure and has been monitored in an outpatient clinic (Supplementary Figures S2, S3).

## Discussion

More than 60% patients with vEDS tend to present life-threatening vascular complications before the age of 40 (7). Various disastrous manifestations are possible involving multiple different organs, including medium or small arteries, bowel, and uterus. While the majority of studies reported one-off events, metachronous lesions of bilateral arteries are rarely reported (8, 9). This patient was affected by dissecting aneurysm of right iliac artery, followed by left ilio-iliac AVF 3 months later, and underwent urgent operations.

The patient is about 181 cm tall and 85 kg (BMI 25.9) with a medium build. In retrospect, easy bruising and lucent skin with vascular visibility and tooth decay have been noted since childhood. The patient also had prominent eyes and a long thin nose. Even during postoperative period, periorbital ecchymosis without any trauma was observed. However, no other physical characteristics, such as skin hyper-extensibility or delayed wound healing, typical of classic EDS were found.

In most patients, vEDS is likely to be suspected based on family history. The patient is Korean, with both parents of Korean descent. The patient's father served in the Vietnam War and died of lung cancer, presumably caused by defoliants when he was 10 years old. His mother had no signs of structural vulnerability and the patient is an only son without siblings. Although the final diagnosis suggested a paternal history of autosomal dominant vEDS, *de novo* mutations cannot be excluded considering that half of the probands also reported no family history of vEDS (2).

Since the publication of Ehlers-Danlos syndromes: 1997 Villefranche classification by Ehlers-Danlos National Foundation (USA) and Ehlers-Danlos Support Group (UK), the clinical diagnosis of specific EDS subtypes was based on biochemical and molecular features. The 2017 revised international EDS consortium guidelines recommended molecular screening for *COL3A1* and *COL1A1* genes to establish a diagnosis of vEDS in individuals with a family history of vEDS, arterial rupture or dissection in those under the age of 40 years, unexplained sigmoid colon rupture, or spontaneous pneumothorax in the presence of other characteristics consistent with vEDS (10). To exclude other diseases of similar manifestation such as Loeys-Dietz syndrome (LDS) and Marfan syndrome (MFS), we performed a targeted sequencing of genes including *SMAD2, SMAD3, TGFB3, TGFBR1*, and *TGFBR2* as well as *COL3A1* and *COL1A1*. This patient had no typical skeletal deformities of MFS or LDS but AVF was occasionally reported in such diseases. No pathogenic variants were detected other than *COL3A1*. Therefore, no other approaches were necessary such as copy number variation (CNV) detection or sodium dodecyl sulfate polyacrylamide gel electrophoresis (SDS-PAGE) for the identification of protein deletions or duplications.

Despite the rarity of the disease, Eagleton et al. suggested a few general guidelines for open repair, including the use of padded vascular clamps, felt reinforcement or tissue sealants along the suture line, and interrupted sutures to prevent unnecessary traumatic injury to friable vessels (11). While traditionally open approaches with primary vessel repair using pledgets are preferred, the endovascular method has been increasingly utilized as appropriate following the development of minimally invasive techniques (12, 13). However, no noticeable improvement was found in endovascular outcomes, except for a small reduction in mortality (13). Additionally, percutaneous arterial access in vEDS can precipitate multiple bleeding complications such as femoral artery rupture or pseudoaneurysms, especially when large devices are used (13, 14). Special attention has already been emphasized to reduce the risk of device-induced or procedure-related vessel damage (15). In the first operation, as mentioned in literature, felt pledgets were used to anastomose the right femoral artery with the graft following the failure of percutaneous closing device. The femoro-femoral bypass was not performed to avoid additional incisions and vascular manipulation of other sites considering the condition of the pathological vessel and possible future events, although no vEDS was diagnosed at the time. In the second operation, we initially determined to incise the groin for surgical repair following endovascular AVF exclusion. The access artery was meticulously sutured in an interrupted manner without felt pledgets, unlike the first operation. Adjusting the force without damaging the friable vessels is more important than using the felt. No adverse events were induced by the overlapped stent-grafts at both access sites. Stent-graft insertion and access artery repair after cutdown is safer than a completely percutaneous or open surgical approach, which was already recommended by Brooke et al. in patients with MFS (16). The use of endovascular closing devices for access sites should be avoided if possible.

In the second operation for ilio-iliac AVF, a self-expanding stent rather than a covered stent was used to cover AVF opening and insert coils precisely by increasing the friction between IIA and coils, especially in the proximal IIA with a short length (5 mm) before fistula. However, we failed to cover the AVF completely and a piece of coil entered the blood flow through the AVF opening, which increased the risk of postoperative DVT below the left CIV. It was imperative to achieve complete orifice coverage through the addition of a supplementary stent, coupled with a heightened focus on precise execution during coil embolization considering the high flow rate.

The insufficient tensile strength of the vessel wall in patients with vEDS can hamper surgical procedures, resulting in potentially fatal outcomes. Therefore, it is more important to suspect the diagnosis and consider every possible precaution in extreme scenarios preoperatively. Not surprisingly, the incidence of perioperative complications such as frequency of open repair and intraoperative mortality was higher during emergency surgical procedures without diagnostic information (17). However, it is hard to determine the condition of blood vessels based on images alone. Even in this single patient, attitudes toward the first and second operations appeared to differ depending on whether a pathological condition was suspected. Weak and fragile vessels are a surgical challenge, as in the first operation of this case. However, based on the initial experience of tissue fragility, adequate precaution was exercised during the second intervention. Likewise, suspicion based on knowledge of the disease is important as it can provide early insights. Although the rarity of the disease and the unpredictability of events makes it difficult to conduct randomized controlled trial, this case, similar to other uncommon diseases, provides insight into the natural history and treatment of vEDS.

Once vEDS is diagnosed, the patients should be under strict multi-disciplinary care. Extra attention is needed to prevent the risk of bleeding due to vascular fragility. Life-style modification such as avoiding collision sports and isometric activities is necessary to reduce the chance of traumatic injury. Meanwhile, medical management of vEDS has been reported to prevent catastrophic results (18, 19). The major goal of medical management is to maintain normal or low blood pressure to avoid arterial dissection or rupture (2). This patient is also undergoing treatment with a beta-blocker.

Fortunately, this patient had no vascular events or bleeding complications after the second operation despite anticoagulant therapy for about a year. However, as in this case, a dilemma arises concerning the appropriate utilization of antithrombotics for patients with vEDS who develop DVT or had been treated with endovascular prosthesis.

## Conclusion

Once vEDS is suspected in young patients with unexpected non-traumatic arterial events, careful planning before operations and vigilance during procedures are crucial for managing unforeseen events. Given the vascular fragility and the potential for future events, it is recommended to refrain from employing additional vascular manipulation unless it is in a life-threatening condition. In particular, meticulous hybrid interventions can be effective treatments.

## Data Availability

The datasets presented in this article are not readily available because the genetic test was carried out by delegating it to an external professional entity. Requests to access the datasets should be directed to db.surlife@gmail.com.

## Supplementary material

Supplementary Figure S1 The 30-year-old male patient has thin and translucent skin with remarkably increased venous visibility on the right flank.

Supplementary Figure S2 The follow-up CT scan 14 months after the 2nd operation shows well-placed stent-grafts and both groins without any abnormality. Left internal iliac artery is also well occluded. (**A**) RAO 30 (**B**) LAO 30.

Supplementary Figure S3 Timeline of clinical events (orange, emergency room (ER) visit; green, diagnosis of deep vein thrombosis (DVT); blue, abdominal computed tomography (CT) scans; black, duplex scans for DVT). The diagnosis of vascular Ehlers-Danlos Syndrome was confirmed through genetic testing following the occurrence of two subsequent events involving bilateral iliac arteries. Follow-up CT scans conducted on postoperative day 10 and 14 months later revealed no evidence of active bleeding or arterial abnormalities. Regarding postoperatively developed proximal DVT in bilateral legs, the patient was prescribed Rivaroxaban (Xarelto®) for anticoagulation therapy. Duplex scans were performed every 3 months to monitor progress. The initial dose was 20 mg once daily for the first three months following the diagnosis of bilateral proximal DVT. Subsequently, the dose was reduced to 15 mg once daily for the next three months and further decreased to 10 mg once daily. The decision to extend the anticoagulation period was made due to the persistence of proximal leg DVT, although there was a significant reduction in its severity. After a total duration of slightly over a year of anticoagulation, the therapy was discontinued, instead Aspirin 100 mg once daily was prescribed. The patient is now scheduled for periodic outpatient clinic follow-ups. Additionally, the patient has been treated with a beta-blocker (Carvedilol) to manage blood pressure since February 2023.
