# Supplementary figures and images for: Case Report: Hybrid approach as a Rescue Treatment in a patient with vascular Ehlers–Danlos Syndrome
Source: Front Surg. 2023 Oct 23;10:1268671. doi: 10.3389/fsurg.2023.1268671 (PMC10627186; doi:10.3389/fsurg.2023.1268671)

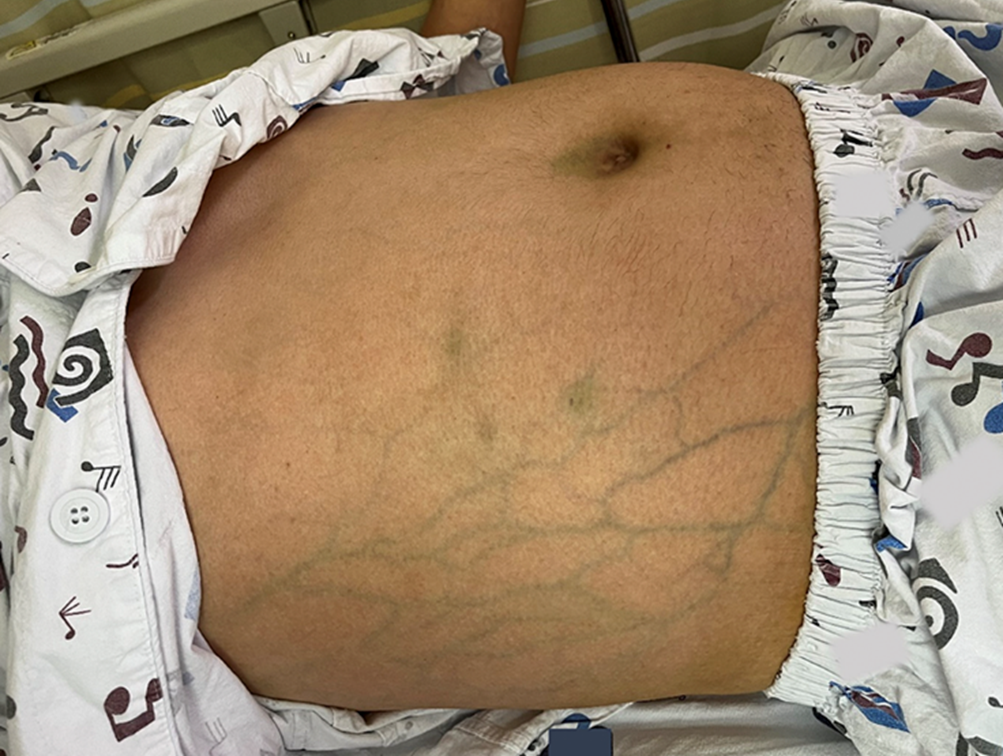

Supplement: Supplementary Figure S1 — The 30-year-old male patient has thin and translucent skin with remarkably increased venous visibility on the right flank. [file Image1.tif]

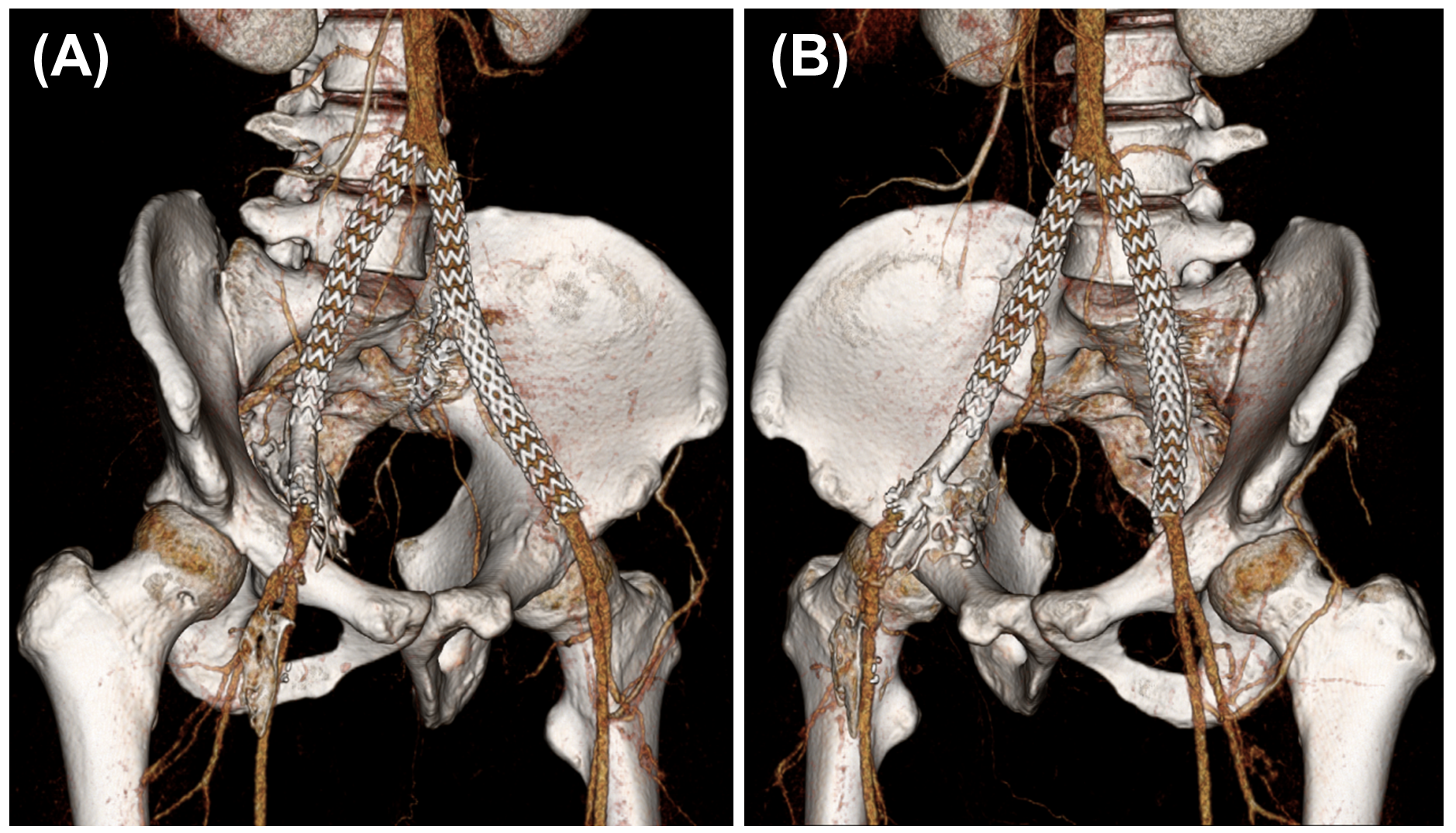

Supplement: Supplementary Figure S2 — The follow-up CT scan 14 months after the 2nd operation shows well-placed stent-grafts and both groins without any abnormality. Left internal iliac artery is also well occluded. (A) RAO 30 (B) LAO 30. [file Image2.tif]

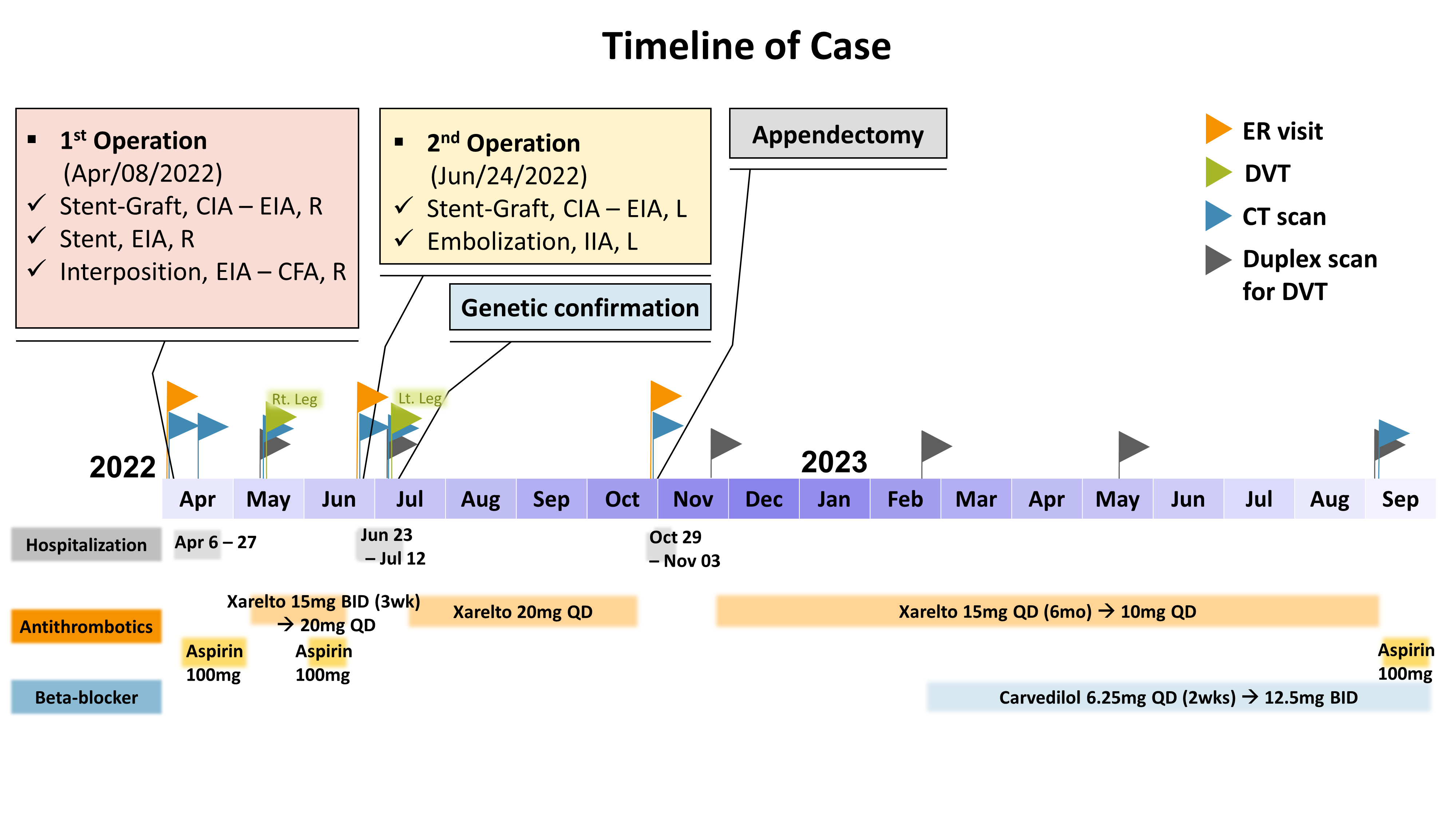

Supplement: Supplementary Figure S3 — Timeline of clinical events (orange, emergency room (ER) visit; green, diagnosis of deep vein thrombosis (DVT); blue, abdominal computed tomography (CT) scans; black, duplex scans for DVT). The diagnosis of vascular Ehlers-Danlos Syndrome was confirmed through genetic testing following the occurrence of two subsequent events involving bilateral iliac arteries. Follow-up CT scans conducted on postoperative day 10 and 14 months later revealed no evidence of active bleeding or arterial abnormalities. Regarding postoperatively developed proximal DVT in bilateral legs, the patient was prescribed Rivaroxaban (Xarelto®) for anticoagulation therapy. Duplex scans were performed every 3 months to monitor progress. The initial dose was 20 mg once daily for the first three months following the diagnosis of bilateral proximal DVT. Subsequently, the dose was reduced to 15 mg once daily for the next three months and further decreased to 10 mg once daily. The decision to extend the anticoagulation period was made due to the persistence of proximal leg DVT, although there was a significant reduction in its severity. After a total duration of slightly over a year of anticoagulation, the therapy was discontinued, instead Aspirin 100 mg once daily was prescribed. The patient is now scheduled for periodic outpatient clinic follow-ups. Additionally, the patient has been treated with a beta-blocker (Carvedilol) to manage blood pressure since February 2023. [file Image3.tif]
